# Entity recognition in Chinese clinical text using attention-based CNN-LSTM-CRF

**DOI:** 10.1186/s12911-019-0787-y

**Published:** 2019-04-04

**Authors:** Buzhou Tang, Xiaolong Wang, Jun Yan, Qingcai Chen

**Affiliations:** 1 Key Laboratory of Network Oriented Intelligent Computation, Harbin Institute of Technology, (Shenzhen), Shenzhen, 518055 China; 2 Yidu Cloud (Beijing) Technology Co., Ltd, Beijing, 100191 China

**Keywords:** Chinese clinical entity recognition, Neural network, Convolutional neural network, Long-short term memory, Conditional random field

## Abstract

**Background:**

Clinical entity recognition as a fundamental task of clinical text processing has been attracted a great deal of attention during the last decade. However, most studies focus on clinical text in English rather than other languages. Recently, a few researchers have began to study entity recognition in Chinese clinical text.

**Methods:**

In this paper, a novel deep neural network, called attention-based CNN-LSTM-CRF, is proposed to recognize entities in Chinese clinical text. Attention-based CNN-LSTM-CRF is an extension of LSTM-CRF by introducing a CNN (convolutional neural network) layer after the input layer to capture local context information of words of interest and an attention layer before the CRF layer to select relevant words in the same sentence.

**Results:**

In order to evaluate the proposed method, we compare it with other two currently popular methods, CRF (conditional random field) and LSTM-CRF, on two benchmark datasets. One of the datasets is publically available and only contains contiguous clinical entities, and the other one is constructed by us and contains contiguous and discontiguous clinical entities. Experimental results show that attention-based CNN-LSTM-CRF outperforms CRF and LSTM-CRF.

**Conclusions:**

CNN and attention mechanism are individually beneficial to LSTM-CRF-based Chinese clinical entity recognition system, no matter whether contiguous clinical entities are considered. The conribution of attention mechanism is greater than CNN.

## Introduction

With rapid development of electronic medical information systems, more and more electronic medical records (EMRs) are available for medical research and application. In EMRs, plenty of useful information is embedded in clinical text. The first step to use clinical text is clinical entity recognition that finds which words form clinical entities and which type each entity belongs to.

In the last decades, a large number of methods have been proposed for clinical entity recognition. The methods includes early rule-based methods, machine learning methods based on manually-crafted features in past a few years and recently deep neural networks. The most popular machine learning method used for clinical entity recognition is conditional random field (CRF) [1], and the most popular deep neural network is LSTM-CRF [2]. However, most studies focus on entity recognition in English clinical text rather than other languages. It is necessary to investigate the latest methods for entity recognition in other languages, for example Chinese.

To promote development of entity recognition in Chinese clinical text, the organizers of China conference on knowledge graph and semantic computing (CCKS) launched a challenge was launched in 2017 [3]. The challenge organizer provided a dataset (called CCKS2017_CNER) with only contiguous clinical entities following the guideline of i2b2 (Informatics for Integrating Biology and the Bedside) challenge for English clinical text in 2010 [4]. Nearly all systems proposed for CCKS2017 challenge adopted CRF or LSTM-CRF. In addition, discontiguous clinical entities composed of discontiguous words, accounting for around 10% in English clinical text, also widely exist in Chinese clinical text. No study have ever considered discontiguous entities in Chinese clinical text.

In this study, we propose a novel deep neural network, called attention-based CNN-LSTM-CRF, for entity recognition considering both contiguous and discontiguous entities in Chinese clinical text. Attention-based CNN-LSTM-CRF is an extension of LSTM-CRF by adding two layers. A dataset (called ICRC-CNER) containing both Chinese contiguous and discontiguous entities is constructed by us (the intelligence computing research center (ICRC) of Harbin institute of technology, Shenzhen) and used to evaluate attention-based CNN-LSTM-CRF. Experiments conducted on CCKS2017_CNER and ICRC_CNER show that our proposed method outperforms CRF and LSTM-CRF. It should be stated that this paper is an extension of our previous paper [5].

### Related work

Clinical entity representation is very important for recognition. As there exist contiguous and discontiguous entities in clinical text, we could not adopt named entity representation in the newswire domain directly for clinical entities. In order to represent contiguous and discontiguous clinical entities in a unified schema, Tang et al. [6, 7] extended the schemas, such as “BIO” and “BIOES” by introducing new labels for contiguous word fragment shared by discontiguous clinical entities or not, that are “BIOHD” and “BIOHD1234”. Wu et al. [8] proposed a schema, called “Multi-label” to give each word multiple labels, each one of which corresponds the label of the token in one clinical entities.

In the past several years, as a number of manually annotated corpora have been publically available for clinical entity recognition in challenges such as the Center for Informatics for Integrating Biology & the Beside (i2b2) [4, 9–11], ShARe/CLEF eHealth Evaluation Lab (SHEL) [12, 13], SemEval (Semantic Evaluation) [14–17], etc., lots of machine learning methods, such as support vector machine (SVM), hidden markov model (HMM), conditional random field (CRF), structured support vector machine (SSVM) and deep neural networks, have been applied to clinical named entity recognition. Among these methods, CRF is the most frequently used method whole performance relies on manually-crafted features, whereas deep neural networks, especially LSTM-CRF, which have ability to avoid feature engineering, are recently introduced for clinical entity recognition. Common features, such as N-grams and part-of-speech, and domain-specific features, such as section information and domain dictionaries, are usually adopted in CRF. For LSTM-CRF, there are a few variants such as [18, 19], which extend the basic LSTM-CRF by introducing character-level word embeddings or attention mechanism.

## Methods

**Fig. 1:**
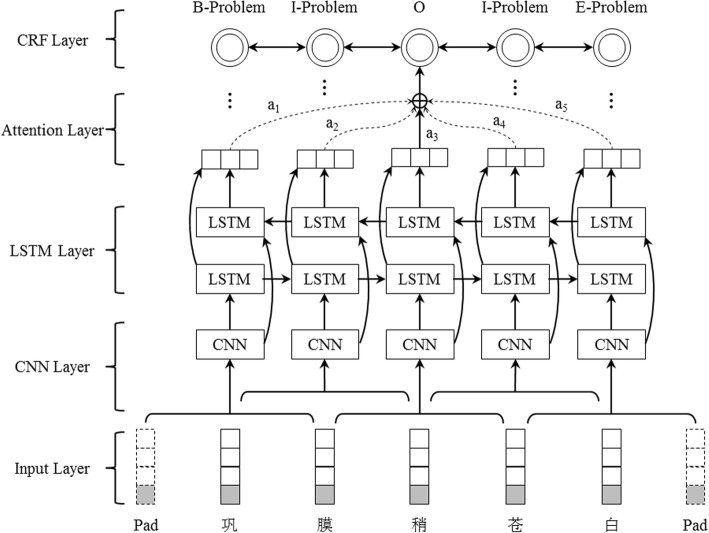
Overview architecture of attention-based CNN-LSTM-CRF

The overview architecture of attention-based CNN-LSTM-CRF is shown in Fig. 1. It consists of the following five layers: 1) Input layer, which takes the representation of each Chinese character in a sentence; 2) CNN layer, which represents the local context of a Chinese character of interest within a sliding window (e.g. [− 1, 1] in Fig. 1); 3) LSTM layer, which uses a forward LSTM and a backward LSTM to model a sentence to capture global context information of a sentence; 4) Attention layer, which determines relativity strength of other Chinese characters to a Chinese character of interest; 5) CRF layer, which predicts a label sequence for an input sentence by considering relations between neighbor labels. The five layer is presented in detail in the following sections.

### Input layer

As we all know, Chinese text processing is different from English text processing as there is no separator between words. Therefore, word segmentation is usually a first step for Chinese text processing. However, there is no publicly available Chinese word segmentation tool in the clinical domain, and Chinese word segmentation tools developed in other domains have been proved detrimental to Chinese clinical entity recognition [20]. Therefore, in this study, Chinse clinical sentences are segmented into single Chinese characters as shown in Fig. 1 (“巩膜稍苍白” – “slight pallor of the sclera” was segmented into “巩”, “膜”, “稍”, “苍”, “白”.).

Formally, given a Chinese clinical sentence *s* = *w*_0_*w*_1_…*w*_*n*_, where *w*_*t*_ (1 ≤ *t* ≤ *n*) is the *t*-th Chinese character, we follow the previous study [21] to represent *w*_*t*_ by *x*_*t*_ = [*c*_*wt*_; *r*_*wt*_], where *c*_*wt*_ and *r*_*wt*_ are embeddings of *w*_*t*_ and its radical respectively, and ‘;’ is the concatenation operation.

### CNN layer

**Fig. 2:**
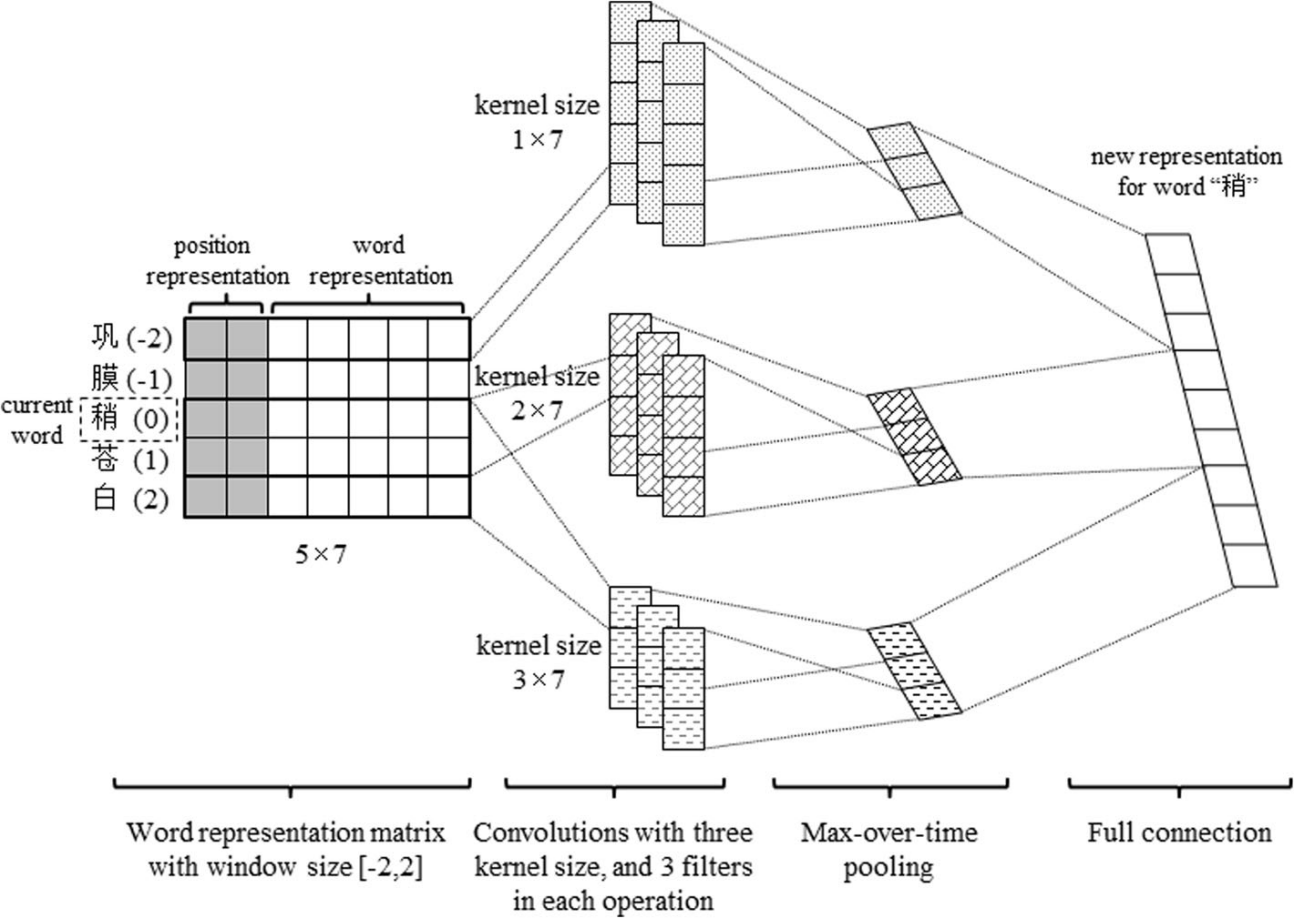
Overview architecture of the CNN layer

Convolutional neural network (CNN), as shown in Fig. 2, is employed to extract local context information of a Chinese character of interest in the following four steps:

1. Input matrix. The context of *w*_*t*_ within a window of [−*m*, *m*], *w*_*t-m*_…*w*_*t + m*_, is represented by *Q* = [[*x*_*t-m*_; *p*_-*m*_], …, [*x*_*t + m*_; *p*_*m*_]], where *p*_*i*_ (−*m* ≤ *i* ≤ *m*) is position embeddings for the distance of *w*_*i*_ relative to *w*_*t*_.
2. Convolution operation. Convolution kernels of different size *M* are employed for feature extraction. Suppose that there are *L* filters (feature maps) for each size, let *W*^(*u*, *v*)^ (1 ≤ *u* ≤ *M*, 1 ≤ *v* ≤ *L*) denotes the *v*-th filter of size *u*. Then, the following convolution operation is applied on *Q*:

1 $$ {F}_i^{\left(u,v\right)}=\sigma \left({W}^{\left(u,v\right)}\otimes {Q}_{\left[i:i+k-1\right]}+{b}^{\left(u,v\right)}\right)\ \left(1\le i\le m-k+1\right) $$

where ${F}_i^{\left(u,v\right)}$ is the *i*-th feature extracted from context matrix *Q* by filter *W*^(*u*, *v*)^, *σ* is the element-wise sigmoid function, ⊗ is the element-wise product, and *b*^(*u*, *v*)^ is a bias vector. All features extracted by filter *W*^(*u*, *v*)^ can be represented as ${F}^{\left(u,v\right)}=\left({F}_1^{\left(u,v\right)},{F}_2^{\left(u,v\right)},\dots, {F}_{m-k+1}^{\left(u,v\right)}\right)$.

3) Max-pooling operation. After the convolution operation, a max-over-time pooling operation is employed on each filter to select the most significant feature as follows:

2 $$ {F}_{max}^{\left(u,v\right)}=\max \left\{{F}_1^{\left(u,v\right)},{F}_2^{\left(u,v\right)},\dots, {F}_{m-k+1}^{\left(u,v\right)}\right\} $$

Until now, the features corresponding to convolution kernels of size *u* are ${F}^{(u)}=\left({F}_{max}^{\left(u,1\right)},{F}_{max}^{\left(u,2\right)},\dots, {F}_{max}^{\left(u,L\right)}\right)$.

4) Full connection. Finally, all features outputted after max-pooling are concatenated together to represent the local context of *w*_*t*_, that is ${g}_t=\left({F}_t^{(1)};{F}_t^{(2)};\dots; {F}_t^{(M)}\right)$.

After the CNN layer, the sentence representation becomes *g* = (*g*_1_, *g*_2_,  … , *g*_*n*_).

### LSTM layer

Taking *g* = (*g*_1_, *g*_2_,  … , *g*_*n*_) outputted by the CNN layer as input, the LSTM layer produces a new representation sequence *h* = (*h*_1_, *h*_2_,  … , *h*_*n*_), where *h*_*t*_ = [*h*_*ft*_; *h*_*bt*_] (1 ≤ *t* ≤ *n*) concatenates the outputs of both forward LSTM *h*_*ft*_ and backward LSTM *h*_*bt*_ at step *t*. An LSTM unit is composed of one memory cell and three gates (input gate, forget gate and output gate), denoted by *c*_*t*_, *o*_*t*_, *i*_*t*_ and *f*_*t*_ respectively for the LSTM unit at step *t*. Taking *g*_*t*_, *h*_*t* − 1_, *c*_*t* − 1_ as input at step *t*, the LSTM unit can produce *h*_*t*_ and *c*_*t*_ as follows:

3 $$ {\displaystyle \begin{array}{c}{i}_t=\sigma \left({W}_{gi}{g}_t+{W}_{hi}{h}_{t-1}+{W}_{ci}{c}_{t-1}+{b}_i\right)\\ {}{f}_t=\sigma \left({W}_{gf}{g}_t+{W}_{hf}{h}_{t-1}+{W}_{cf}{c}_{t-1}+{b}_f\right)\\ {}{c}_t={f}_t\otimes {c}_{t-1}+{i}_t\otimes \mathit{\tanh}\left({W}_{gc}{g}_t+{W}_{hc}{h}_{t-1}+{b}_c\right)\\ {}{o}_t=\sigma \left({W}_{go}{g}_t+{W}_{ho}{h}_{t-1}+{W}_{co}{c}_t+{b}_o\right)\\ {}{h}_t={o}_t\otimes \mathit{\tanh}\left({c}_t\right)\end{array}} $$

where *σ* is the element-wise sigmoid function, ⊗ is the element-wise product, *W*_*i*_, *W*_*f*_, *W*_*c*_ and *W*_*o*_ (with subscripts: *g*, *h* and *c*) are the weight matrices, *b*_*i*_, *b*_*f*_, *b*_*c*_ and *b*_*o*_ are bias vectors.

### Attention layer

**Fig. 3:**
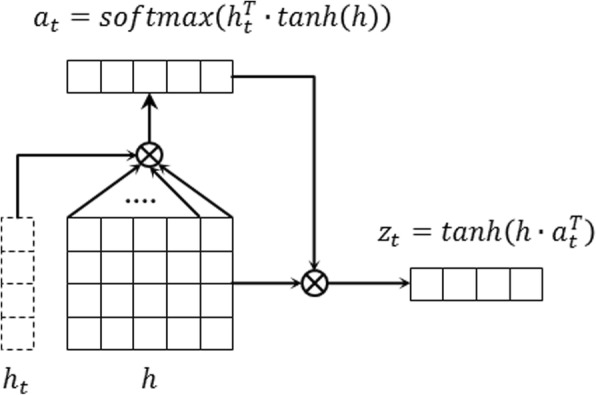
Overview architecture of the attention layer

An attention network, as shown in Fig. 3, is employed to determine relativity strength of other Chinese characters to the Chinese character of interest, under the assumption that the label of *w*_*t*_ is not determined by *h*_*t*_ only. For example, in a fragment “皮肤粗糙、苍白” (“hard and pale skin”), “皮肤粗糙” (“hard skin”) is a contiguous problem, and “皮肤…苍白” (“pale skin”) is a discontiguous problem with two words “皮肤” (“skin”) and “苍白” (“pale”). The word “皮肤” is not a clinical entity only when it appears with word “苍白”, which means that the label of word “皮肤” also depends on the word “苍白”.

Taking the representation sequence *h* outputted by the LSTM layer as input, the attention layer produces a new representation sequence *z* = (*z*_1_, *z*_2_,  … , *z*_*n*_), where *z*_*t*_ at step *t* can be calculated as follows:

4 $$ {z}_t=\mathit{\tanh}\left(h\cdotp {a}_t^T\right) $$

where *tanh* is the activation function, *h* is the representation matrix outputted by LSTM layer, *a*_*t*_ is the weight vector for each word in the sentence calculated as follows:

5 $$ {a}_t= softmax\left({h}_t^T\cdotp \mathit{\tanh}(h)\right) $$

where *softmax* is the normalization function, *h*_*t*_ is the representation of *h* at step *t*. Finally, the new representation sequence *z* is applied for the label prediction in the next CRF layer.

### CRF layer

The CRF layer takes sequence *z* = (*z*_1_, *z*_2_,  … , *z*_*n*_) as input, and predicts the most possible label sequence *y* = (*y*_1_, *y*_2_,  … , *y*_*n*_). Give a training set *D*, all parameters of CRF layer (denoted as *θ*) are estimated by maximizing the following log-likelihood:

6 $$ L\left(\theta \right)=\sum \limits_{\left(s,y\right)\in D}\mathit{\log}p\left(y|z,\theta \right) $$

where *y* is the corresponding label sequence of sentence *s*, *p* is the conditional probability of *y* when given *s* and *θ*. Assuming that *S*_*θ*_(*z*, *y*) is the score of label sequence *y* for sentence, then the conditional probability *p* can be calculated as the normalization of *S*_*θ*_(*z*, *y*). In order to take full advantage of dependencies between neighbor labels, the model incorporates a transition matrix *T* with an emission matrix *E* to calculate the score of label sequence *S*_*θ*_(*z*, *y*), as follows:

7 $$ {S}_{\theta}\left(z,y\right)=\sum \limits_{t=1}^n\left({E}_{y_t,t}+{T}_{y_{t-1},{y}_t}\right) $$

where ${E}_{y_t,t}$ is the probability that word *z*_*t*_ with label *y*_*t*_, and ${T}_{y_{t-1},{y}_t}$ is the probability that word *z*_*t* − 1_ with label *y*_*t* − 1_ followed by *z*_*t*_ with label *y*_*t*_. We can maximize the log-likelihood (6) over all training set *D* by the dynamic programing, and find the best label sequence for any input sentence by maximizing score (7) using Viterbi algorithm.

### Dataset

We evaluate the attention-based CNN-LSTM-CRF on two datasets: CCKS2017_CNER and ICRC_CNER. CCKS2017_CNER contains 400 Chinese clinical records with five categories of clinical entities, 300 records are treated as a training set and the remainder 100 records are treated as a test set. In this dataset, all clinical entities are contiguous, and the total number of them is 39,359. ICRC_CNER contains 1176 Chinese clinical records with the other five categories of clinical entities, 600 records are treated as a training set, 176 records are treated as a development set and the remainder 400 records are treated as a test set. In this dataset, both contiguous and dis contiguous clinical entities are manually annotated, and the total number of clinical entities is 91,185. Table 1 list the statistics of the two datasets, where “#*” denotes the number of “*”, and the numbers of contiguous entities and discontiguous entities in ICRC_CNER are given in separated rows (the numbers of contiguous entities in the upper rows, and the number of discontiguous entities in the lower rows).

**Table 1:** Statistics of CCKS2017_CNER and ICRC_CNER for entity recognition in Chinese clinical text

Dataset (CCKS2017_CNER)	#Record	#Clinical Entity					
		#Body	#Disease	#Symptom	#Test	#Treament	#All
Training (300)	300	10,719	722	7831	9546	1048	29,866
Test (100)	100	3021	553	2311	3143	465	9493
Total (400)	400	13,740	1275	10,142	12,689	1513	39,359
Dataset (ICRC_CNER)	#Record	#Clinical Entity					
		#Medication	#Disease	#Symptom	#Test	#Treament	#All
Training	600	1293	11,470	5270	17,024	3065	38,122
		0	7441	75	7	107	7630
Development	176	475	3594	1738	5276	938	12,021
		0	2421	37	3	41	2502
Test	400	999	7932	3353	11,326	2020	35,630
		3	5153	57	6	61	5280
Total	1176	2767	22,996	10,361	33,626	6023	75,773
		3	15,015	169	16	209	15,412

### Evaluation and experiments setup

We start from two baseline methods: CRF and LSTM-CRF, then investigate the effects of the CNN layer and attention layer respectively, and finally compare attention-based CNN-LSTM-CRF with other state-of-the-art systems on CCKS2017_CNER. Following previous studies [7, 17], clinical entities in CCKS2017_CNER are represented by “BIO”, and that in ICRC_CNER are represented by “BIOHD1234” and “Multi-label” respectively. The features utilized in CRF are the same as [21], including bag-of-words, part-of-speech, radical information, sentence information, section information, general NER, word representation, dictionary feature, etc. It should be stated that LSTM-CRF here is the same as that used in the best system of CCKS 2017 [21].

The performances of all systems are measured by micro-averaged precision, recall and F1-score under two criteria: “strict” and “relaxed”, where the “strict” criterion checks whether predicted entities exactly match with gold ones in boundary and category, while the “relaxed” criterion relaxes the condition in boundary, and only checks whether predicted entities overlap with gold ones. The “strict” measures are the primary measures.

The hyper-parameters used in LSTM-CRF and attention-based CNN-LSTM-CRF are: dimension of Chinese character embeddings-50, dimension of radical embedding-25, dimension of position embedding-20, size of convolution kernels in the CNN layer-1/2/3, number of filters of each size-32, size of LSTM unit-100, size of sliding window-[− 2,2], dropout probability-0.5 and training epochs-30. The Chinese character embeddings are pre-trained by the word2vec tool (https://github.com/tensorflow/tensorflow/tree/r1.1/tensorflow/examples/tutorials/word2vec) on a large unlabeled dataset provided by CCKS2017, and the radical embeddings are randomly initialized. The parameters of all deep neural network models are estimated using stochastic gradient descent (SGD) algorithm.

## Results

Table 2 shows the performances of different methods on CCKS2017_CNER and ICRC_CNER, where the highest measures are in bold (the following sections also use the same way to denote the highest measures), and the performances of each method using “BIOHD1234” and “Multi-label” on ICRC_CNER are listed in separated rows (the performance measures in the upper rows correspond to “BIOHD1234”, and the performance measures in the lower rows correspond to “Multi-label”). Our method achieves highest “strict” F1-scores of 90.61% on CCKS2017_CNER and 83.32% on ICRC_CNER, outperforming CRF and LSTM-CRF by 0.44 and 0.32% respectively. All methods using “Multi-label” shows better performance than that using “BIOHD1234”.

**Table 2:** Performances of different methods on the two datasets: CCKS2017_CNER and ICRC_CNER

Dataset	Method	Strict (%)			Relaxed (%)		
		Precision	Recall	F1-score	Precision	Recall	F1-score
CCKS2017_CNER	CRF	**91.22**	88.20	89.69	**95.73**	92.57	94.13
	LSTM-CRF	90.68	89.67	90.17	95.18	94.12	94.65
	Our Method	90.73	**90.49**	**90.61**	94.84	**94.59**	**94.71**
ICRC_CNER	CRF	81.84	78.86	80.32	93.75	90.34	92.01
		83.42	79.90	81.62	**94.02**	90.05	92.00
	LSTM-CRF	**83.55**	82.26	82.90	93.80	92.35	93.07
		82.71	83.30	83.00	92.77	93.42	93.09
	Our Method	82.96	82.60	82.78	93.30	92.90	93.10
		82.66	**83.99**	**83.32**	92.57	**94.07**	**93.31**

Table 2 shows the performances of different methods on CCKS2017_CNER and ICRC_CNER, where the highest measures are in bold (the following sections also use the same way to denote the highest measures)

In order to investigate effects of the CNN layer and attention layer in our method respectively, we remove one or two of them from attention-based CNN-LSTM-CRF, and present the results in Table 3, where only precisions, recalls and F1-scores under the “strict” criterion are listed, “w/o” denotes “without”, and our method without both CNN layer and attention layer just becomes LSTM-CRF. When the CNN layer is removed from our method, the F-score slightly increases on CCKS2017, but slightly decreases on ICRC_CNER. When the attention layer is removed, the F-scores on both two datasets decreases slightly. When both CNN and attention layers are removed, the F-scores on both two datasets decreases greatly. The experimental results indicates that both CNN and attention layers are individually beneficial to LSTM-CRF, the contribution of attention layer is greater than CNN layer, but they may hurt each other some times. It may be because contiguous entities only depend on neighbor Chinese characters which are captured by the CNN layer and attention layer repeatedly, whereas discontiguous entities depend on skipping words which may benefit from the attention layer.

**Table 3:** Effects of the CNN layer and attention layer in our method

Method	CCKS2017_CNER (%)			ICRC_CNER (%)		
	Precision	Recall	F1-score	Precision	Recall	F1-score
Our method	90.73	**90.49**	90.61	82.66	**83.99**	**83.32**
w/o CNN	**91.11**	90.48	**90.79**	**83.72**	82.53	83.12
w/o attention	90.61	90.23	90.42	83.16	83.29	83.22
w/o both	90.68	89.67	90.17	82.71	83.30	83.00

Table 2 shows the performances of different methods on CCKS2017_CNER and ICRC_CNER, where the highest measures are in bold (the following sections also use the same way to denote the highest measures)

Furthermore, we also compare our method with the best system of the CCKS2017 challenge, which employed several individual methods, such as rule-based method, CRF, LSTM-CRF without additional features and LSTM-CRF with additional features (the same as the baseline method LSTM-CRF used in this paper), and further used a voting method to integrate all the results of these methods. The best individual method is LSTM-CRF with additional features, which is inferior to attention-based CNN-LSTM-CRF as mentioned above (shown in Table 2). Following the same way to integrate CRF, LSTM-CRF without additional features and our method together, we obtain a “strict” F1-score of 91.46%, higher than that of the best system of the CCKS2017 challenge (i.e., 91.02%) [21].

## Discussion

In order to investigate on which category of clinical entity how our method performs, we list the performance of our method on each category of clinical entity under “strict” criterion in Table 4. Our method performs well on some categories, such as “Test” and “Medication” on ICRC_CNER, “Symptom”, “Test” and “Body” on CCKS2017_CNER dataset. However, it also performs not very well on some categories, such as “Disease” and “Treatment” on both datasets, especially “Symptom” on ICRC_CNER dataset, which is much worse than that on CCKS2017_CNER, may because of a large number of discontiguous clinical entities in “Symptom” category on ICRC_CNER.

**Table 4:** Performances of our CNN-LSTM-Attention model on each category under “strict” criterion

Category	ICRC_CNER (%)			CCKS2017_CNER (%)		
	Pre.	Rec.	F1	Pre.	Rec.	F1
Disease	82.84	81.67	82.25	85.06	77.22	80.95
Symptom	77.06	76.01	76.53	94.92	96.28	**95.60**
Test	84.19	89.03	**86.55**	93.66	93.48	**93.57**
Treatment	77.53	79.58	78.54	77.63	79.08	83.98
Medication	87.88	89.72	**88.79**	/	/	/
Body	/	/	/	86.89	87.36	**87.12**

Table 2 shows the performances of different methods on CCKS2017_CNER and ICRC_CNER, where the highest measures are in bold (the following sections also use the same way to denote the highest measures)

In previous studies, in English clinical text, recognizing discontiguous entities have been proved much more difficult than contiguous entities, and the “strict” F1-score difference on the two types of clinical entities exceededs 25% [21]. However, that difference in Chinese clinical text is around 15% as shown in Table 5. It means that discontiguous entities in Chinese clinical text is much easier than that in English clinical text. Among three method, our method achieves the highest “strict” F1-scores on both two types of clinical entities.

**Table 5:** Performances of methods on contiguous and discontiguous clinical entity under “strict” criterion on ICRC_CNER

Method	Contiguous entity (%)			Discontiguous entity (%)		
	Precision	Recall	F1-score	Precision	Recall	F1-score
CRF	83.52	84.35	83.93	**82.73**	58.26	68.37
LSTM-CRF	83.35	87.35	85.30	78.70	63.62	70.36
Our method	**83.57**	**87.75**	**85.61**	77.17	**65.76**	**71.01**

Table 2 shows the performances of different methods on CCKS2017_CNER and ICRC_CNER, where the highest measures are in bold (the following sections also use the same way to denote the highest measures)

**Fig. 4:**
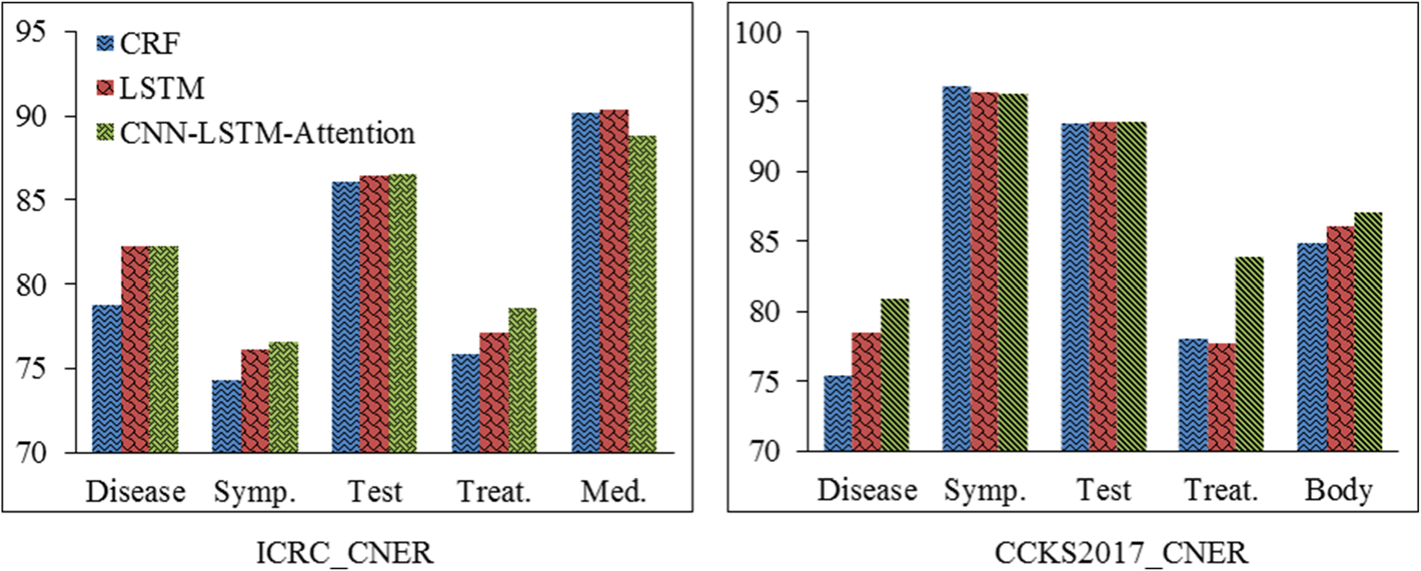
“strict” F1-scores of different methods on each category of clinical text

Although our method shows better overall performance than CRF and LSTM-CRF, it does not always achieve highest “strict” F1-score on all categories of clinical entities. Figure 4 shows the performances of different methods on each category of clinical entity. Our method achieves the highest “strict” F1-scores on all categories except “Medication” on ICRC_CNER and “Symptom” on CCKS2017_CNER. It may be caused by different guidelines. The limitations of this study are: 1) the proposed method is also applicable to entity recognition in English text, but we do not compare it on English datasets. The experiments will be conducted in the future. 2) there also some other extensions of LSTM-CRF on tasks in other domains, we do not compare them with our method in this study. Comparing our method with them and introducing their characteristics into our method to form new methods are other two cases of our future work.

## Conclusions

In this study, we propose a novel deep neural network for entity recognition in Chinese clinical text, which extends LSTM-CRF by introducing a CNN layer and an attention layer. The CNN layer is used to capture local context information of the Chinese character of interest, and the attention layer is used to determine relativity strength of other Chinese characters to the Chinese character of interest. Experiments on two benchmark datasets shows the effectiveness of our proposed method.
